# Billy Sunday

**Published:** 1917-01

**Authors:** 


### Billy Sunday.

Without regard to religious questions, those referring to the sincerity and propriety of Mr. Sunday's expressions, or the ultimate influence of his work, certain points of comparison between the clerical and medical professions are of interest.

This movement is only one of a number indicating the effacement of sectarian lines up to the limit of practical possibility, by religious organizations, quite analogous to similar accomplishments in the medical profession, and in both instances, salutary.

There is a wide-spread opinion, even among men themselves holding firm religious convictions, that religion is popularly held to be an uncertainty, that the clergy exert slight influence in worldly and temporal affairs, and that they are restricted by rather recondite ethical conceptions. As a mere matter of statistics, very nearly 40% of our population is formally connected with religious denominations. This does not include pagan Indians, various heathens and the great body of unattached, nor does it allow, so far as many denominations are concerned, for young children, nor for persons non compos mentis. It is true that religious belief does not always have the practical effect that ought to be expected, but the fact remains that the great majority of our population is "incurably religious," as Dr. Holmes has said.

Offhand, one would say that matters of life and health are only more directly demonstrable than spiritual matters, but that they appeal more directly and immediately to mankind, and that they are of practical interest both to the religious and the irreligious. But actual facts scarcely support these a priori conceptions. We cannot conceive of the need of building a tabernacle for a series of meetings devoted to popular medical lectures, nor of raising a hundred thousand dollars or more in a moderate sized city for this purpose. On the contrary, almost any hall suffices if not too large, the lectures can seldom attract an audience for more than a brief series, even if as usual the audience is not asked to support them financially, and the speakers are lucky to get their expenses. Nor can it be said that this is due to prior information on hygienic and sanitary and medical topics and to a glut of such lectures—on the contrary, the attraction of popular religious meetings exists in spite of these factors for the respective subject.

Judged by crude standards of numbers and financial support, and both by campaigns of short duration and unusual interest and by routine activities, the clerical profession, considered merely as a profession, has an advantage over the medical profession, so great as to deserve attention. Let it be clearly understood that we allude not to matters of personal interest and personal economics, but, in both sides of the comparison, solely to matters involving the welfare of the community. As what may be termed a taxing power, the clergy not only exceeds the medical profession but the political government itself. This test, though not an elevated one, is generally conceded to be the best practical one of vital interest in anything.

Possibly the real reason for this contrast between the two professions is that, notwithstanding the notion that the clergyman is limited by rigid ethical conceptions, and by principle rather than to practical results, he shows a surprising adaptability to circumstances. Can we imagine, for example, a body of medical men, publicly admitting the necessity of turning over some phase of the science and art with which they are engaged, to a technically untrained and unlicensed outsider, not even free from serious adverse criticism? The recent Haiselden case illustrates the same professional difference from a somewhat different standpoint. Instinctively and almost unanimously, the interest of the medical profession was centered, not in the question as to whether a life might have been saved, not in the absolute fact of professional skill and judgement, but in the ethical principles violated by undue publicity. In contrast, note the almost universal tolerance of the notoriety seeker in the pulpit, by the most modest and retiring clergyman as an individual, and by the various church organizations collectively. This tolerance is equally manifested toward occupants of the pulpit whose lack of a theologic degree or of general education, or whose blatant methods make them strictly analogous to the medical quack, and to those who are more comparable to members of the medical profession who exploit their social and professional prestige to secure publicity, and who are not honest enough to engage in open quackery. We recollect, for example, the patient submission of a modest clergyman struggling with the problem of a downtown church whose support was gradually dwindling, to the death-blow given his endeavors by a prominent "brother" with almost unlimited financial support, who hired a hall, employed the widest publicity, and entered into immediate competition with the former. Under all these circumstances, differing widely in detail, the average clergyman whose personal ethics are comparable to those of our profession, asks only whether souls are saved. The physician, on the other hand, does not attach so much importance to the accomplishment of cures as to the ethical means employed. In fact, he goes so far in this direction that it is only with an apology to his ethical code, that he uses such words as saved and cured at all. It should be clearly understood that, in these statements, we are trying merely to present facts, not to condemn the attitude of the medical profession. If any censure is due the medical profession for this placing of ethical methods on a higher plane than practical results, it falls equally on the present writer.

A very significant fact in comparing the two professions is that the term congregation refers to a highly organized body of laity, which not only receives the ministry of the clergyman, but which is regularly called together for instruction, and which is a strong and usually unified power to support such social and religious activities as properly come under the professional control of the priesthood. On the contrary, the clientele of the physician—note the absence of a term applying especially to medicine—even when the physician is a general practitioner and so located that his clientele is almost permanently fixed, means almost nothing more than a general list of persons on whom he may call in a professional capacity and usually not of his own initiative. Only occasionally does intimate friendship between physician and patient, and considerable means on the part of the latter, give an opportunity for the former to direct lay forces toward any worthy object. When such opportunity does present itself, it is so occasional that it is impossible to direct it according to any general professional plan, it is often directed according to personal interests, sometimes unwisely, and sometimes even the factor of selfish interest suggests itself. While the benefits accruing are usually considerable, lay benevolence guided by the medical profession is so uncertain and sporadic that it cannot be compared with the definite and regular support to analogous forms of social betterment guided by the clergy.

It should be distinctly understood that this comparison of two professions justly considered as presenting close analogies in general, is not intended to imply a censure of the medical profession, nor do we see just how the suggested improvements of medical and lay co-operation can be brought about. Comparisons are said to be odious, but they are odious only because they tend to disturb the complacent belief that everything is as it should be and that nothing can be improved.

